# The relationship between personality and cognition in older adults with and without early-onset depression

**DOI:** 10.3389/fpsyt.2024.1337320

**Published:** 2024-07-10

**Authors:** Megan Armstrong, Jack Kaufman, Jeremy Maciarz, Daniel Sullivan, Joseph Kim, Vincent Koppelmans, Scott Langenecker, Sara L. Weisenbach

**Affiliations:** ^1^ Stony Brook University, Stony Brook Medicine, Stony Brook, NY, United States; ^2^ University of Texas Southwestern Medical Center, Dallas, TX, United States; ^3^ McLean Hospital, Mass General Brigham, Belmont, MA, United States; ^4^ Department of Psychiatry, University of Michigan, Ann Arbor, MI, United States; ^5^ Brigham and Women’s Hospital, Mass General Brigham, Boston, MA, United States; ^6^ Department of Psychiatry, Harvard Medical School, Cambridge, MA, United States; ^7^ Department of Psychiatry, University of Utah, Salt Lake City, UT, United States; ^8^ Department of Psychiatry, Ohio State University, Columbus, OH, United States

**Keywords:** personality, cognition, depression, aging, executive functions

## Abstract

**Introduction:**

It is well established that personality traits impact cognition, as certain personality factors are associated with performance in specific cognitive domains. However, the findings on the relationships between the Big Five traits and cognition are mixed. Additionally, few studies have explored these relationships in older adults with a history of depression. The present study aimed to (a) evaluate the impact of the Big Five personality traits in older adults with and without a lifetime history of depression; and (b) test the hypotheses that higher trait neuroticism would correlate negatively with cognitive performance, while openness to experience would correlate positively with cognition.

**Methods:**

The sample consisted of 138 participants between the ages of 55 and 78 (*M* = 65.56, *SD* = 6.36). Sixty-two participants met criteria for current or remitted Major Depressive Disorder, while 76 had no history of depression or other mental health disorders. Participants underwent comprehensive neuropsychological testing. Personality was assessed using the NEO Personality Inventory-Revised (NEO-PI-R), while depression status was determined using the Structured Clinical Interview for DSM-5 (SCID-5). Following a series of Pearson correlations of cognitive variables and the five personality factors, linear regression models were estimated for each significant correlation. Demographic variables (i.e., age, education and sex) were entered in block 1, depression status (never vs. ever) was entered in block 2, and the personality factor score, or sub-facet was entered in block 3.

**Results:**

Neuroticism was not associated with cognitive performance on any outcome measure. The facets *Openness to Feelings* and *Openness to Values* were positively related to phonemic fluency. Further *Openness to Values* was positively related to cognitive flexibility.

**Discussion:**

Our results suggest that older people who are (a) more capable of identifying and understanding their feelings and the feelings of others, and (b) who are more willing to re-examine social, political, and religious values perform stronger on tasks measuring verbal fluency and cognitive flexibility, which are aspects of executive functioning. Interventions that aim to enhance open mindedness in older adults may have a parallel impact on improving executive functioning, though this would need to be examined prospectively.

## Introduction

Personality plays an important role in an individual’s well-being, impacting both mental health and cognition. As some cognitive domains decline with age, this relationship bears increased significance because personality may serve as a protective or risk factor for further cognitive decline (1). Some researchers have found significant relationships between cognitive performance and the Five-Factor Model of personality [i.e., Neuroticism, Extraversion, Openness to Experience, Conscientiousness, Agreeableness (2)] in older adults, though findings are inconsistent. Perhaps the most well-established associations in the literature involve neuroticism and openness to experience. Neuroticism is often negatively correlated with overall cognitive performance, while openness to experience is typically positively correlated with overall cognition.

Neuroticism is characterized by negative affect and a susceptibility to experiencing emotional stress (3, 4) and has been associated with impairment in cognition, in part due to heightened activation of the hypothalamic-pituitary- adrenal (HPA) axis resulting in the release of glucocorticoids (specifically cortisol), which can lead to reductions in memory (5–7). It has been proposed that neuroticism may contribute to longstanding heightened HPA axis activity, resulting in the alteration of brain regions that support memory (3). A study conducted by Da Silva and colleagues (4) also found that stress mediated the relationship between neuroticism and executive functioning (EF) in a sample of older adults.

Many studies find that higher neuroticism is associated with poorer cognitive function across domains (3, 8–10). Some have found that older adults who score higher on measures of neuroticism are at increased risk for the development of dementia due to Alzheimer’s disease at follow-up (11, 12). Graham and Lachman found that neuroticism was negatively associated with working memory and reasoning but not significantly associated with processing speed, reaction time, verbal fluency, inductive reasoning, or episodic memory (9). In contrast, Sutin and colleagues (13) found that neuroticism was associated with poorer performance on tasks of memory, speed-attention-executive, visuospatial ability, numeric reasoning, as well as measures of verbal fluency (11). Boyle and colleagues (3) measured global cognition using the Mini Mental State Examination (MMSE) and executive functioning with the Dementia Rating Scale and Trail Making Tests A & B. They found that neuroticism was significantly associated with global cognitive functioning, but not with executive functioning (4, 13).

Contrary to the evidence that neuroticism is linked to poorer cognition, several studies found small or non-significant correlations between neuroticism and overall performance on cognitive measures (14–16). Hultsch and colleagues (15) included multiple measures of cognition (i.e., fact recall, word recall, story recall, vocabulary, verbal fluency, reading comprehension, working memory, comprehension speed, and semantic speed) yet found little correlation between neuroticism and cognitive functioning in any domain. Booth and colleagues (14) analyzed the relationship between the modified MMSE, Hopkins Verbal Learning Test, Stroop Color-Word Test, Trail Making Test Part B, and the Big Five. They only found a significant relationship between neuroticism and Trails B. Lastly, Jelicic and colleagues (16) failed to find a significant relationship between neuroticism and cognition as measured by the MMSE, letter-digit substitution test, the Stroop Color-Word Test interference trial, Category Fluency, and delayed recall from the Visual Verbal Learning Test. Notably, this study utilized the Dutch version of the Eysenck Personality Questionnaire rather than the NEO-PI to measure personality. In summary, neuroticism has been associated with poorer cognition and is a risk factor for the development of dementia, though the strength of the relationship varies by cognitive domain. Several investigations have also failed to find a significant association between neuroticism and cognition, contesting the robustness of this relationship.

Openness to experience is characterized by (a) a propensity toward seeking novel or intellectual experiences, (b) an appreciation of art and beauty, (c) willingness to reassess political or religious values, and (d) an attunement to emotions (2). The existing literature on the relationship between openness to experience and cognition indicates significant positive associations between openness and fluid intelligence, crystallized intelligence, episodic memory, perceptual speed (17), aspects of executive functioning such as verbal fluency (9, 15, 18), and memory in older adults with mild cognitive impairment (19). Ayyotte and colleagues (20) examined the association between personality traits and neuropsychological test performance in depressed and non-depressed older adults. They found that higher openness to experience was correlated with better scores on Trail Making Test Parts A and B, reflecting better processing speed and attentional shifting, among the depressed group. This was linked to superior performance on a task of working memory among all participants, though it was not associated with performance on measures of phonemic fluency or processing speed and visuomotor association (i.e., Symbol Digit Modalities).

Individuals higher in openness are more likely to engage in cognitively or intellectually stimulating activities throughout their lives, such as taking adult education courses or playing bridge (14). Research has demonstrated that higher educational attainment and participation in certain leisure activities are associated with cognitive reserve, thereby reducing the risk for dementia and slowing the progression of cognitive decline in normal aging (21). This link between openness and engagement in intellectually stimulating activities may be the mechanism behind the positive association between openness and cognitive performance.

Relationships between cognition and the other Big Five factors are equivocal. Higher conscientiousness has been associated with better scores on tasks of memory (10) and executive functioning, as evidenced by performance on Trail Making Test B (22), as well as visuospatial ability, verbal fluency, speed-attention-executive, and numeric reasoning tasks (13). However, these findings contradict several studies that failed to find a significant relationship with working memory, processing speed, reaction time, reasoning, episodic memory, verbal fluency (9), and executive functioning (measured by Trail Making Test B and the Backward Digit Span Task) (20). Similarly, the findings related to extraversion have been domain-specific and largely inconsistent. One study found a negative association between extraversion and reasoning (23). Sutin et al. (13) found a positive correlation between extraversion and performance on speed-attention-executive and fluency tasks, but not on measures of episodic memory, visuospatial ability, or numeric reasoning. Contrasting Sutin et al.’s finding that extraversion predicted better performance on speed-attention-executive tasks (13), other studies found that extraversion was unrelated to aspects of executive functioning (14, 24). Booth and colleagues (14) also failed to find a significant association between extraversion and general cognitive ability or memory.

Finally, significant associations between agreeableness and cognition have been limited. Some studies have been unable to detect a link between agreeableness and cognition, as evidenced by performance on measures of memory (10, 22), attention, executive functioning, language, and visuoperception (22). However, Aiken-Morgan and colleagues (25) found a significant positive association between agreeableness and verbal learning, attention, and working memory. Other studies found negative associations between agreeableness and inductive reasoning and spatial orientation (26) as well as verbal fluency, reasoning, and reaction time (23). Further study is needed to clarify the relationships of extraversion, conscientiousness, and agreeableness with cognition.

Few studies have explored the impact of personality on cognition in older adults with depression. One hypothesized that depression moderates the relationship between neuroticism and cognition in a sample of older adults with late life depression (3). The authors utilized the Mini-Mental State Examination (MMSE), the Initiation- Perseveration subscale of the Mattis Dementia Rating Scale, and trail making tests A and B to assess cognition. They did not find a significant interaction between depression and performance on the MMSE after setting a stringent α = .01 (*p* = .03). Additionally, the neuroticism × depression interactive was marginally significant for Trails A (*p* = .04), but not significant for Trails B or the Initiation-Perseveration subscale of the Mattis Dementia Rating Scale. This study is limited by focusing exclusively on the personality trait neuroticism, and that it did not examine associations for specific cognitive domains.

Another study examining cognitive and personality differences in older adults with a history of early-onset depression, currently remitted (onset prior to age 60) found that participants with a history of depression had significantly lower extraversion scores compared to controls; however, they did not differ on neuroticism (27). The depressed group also showed preserved processing speed, working memory, episodic memory, and overall executive functioning. Importantly, this investigation did not study the interaction between personality and cognition in these samples.

To our knowledge, there have been no studies to date that have focused on the impact of early-onset depression (here defined as onset prior to age 35) and personality on cognition in older adults. Understanding a potential synergistic effect of depression and personality on cognition may help identify individuals at greater risk for cognitive impairment. This may provide valuable insight into the role of personality as a contributor to cognitive reserve or a risk factor for age-related decline in older adults with depression. As such, the aim of this study was to identify the extent to which neuroticism, openness, agreeableness, extraversion, and conscientiousness predict performance across multiple cognitive domains in a sample of older adults. Analyses were adjusted for demographic factors associated with cognitive functioning (i.e., age, education, biological sex) and for depression status (i.e., ever present versus never present). Given that neuroticism (characterized by a propensity toward experiencing chronic stress) is linked to reduced memory and executive functioning, we hypothesized that higher neuroticism in the context of a history of depression would negatively impact cognitive performance on episodic memory and executive functioning. Drawing upon prior research on openness and cognition, we predicted that higher scores in openness will positively predict cognitive performance across all domains.

## Method

### Participants

The present sample was derived from a NIH-funded study that occurred at three different sites in the Mountain West (*n* = 35), Midwest (*n* = 17), and East Coast (*n* = 85) regions of the United States. The parent study investigated the relationship between emotion regulation and cognition in older adults with or without a lifetime history of depression (onset prior to age 35). The present sample included 138 participants between 55 and 78 years of age. **Table 1** displays demographic characteristics for those with current and past histories of depression relative to individuals with no history of depression. Age, *t*(136) = 1.32, *p* = .19, and years of education, *t*(135) = 1.35, *p* = .18, were not significantly different between the depressed and non-depressed groups. Distribution of females and males in each group was not significantly different, χ*^2^* (1, *n* = 79) = 3.61, *p* = .06. As expected, depression symptom severity was higher in those with lifetime history of depression, *t*(134) = -9.59, *p* <.001.

**Table 1:** Sociodemographic Characteristics of Participants.

Baseline characteristic	Controls		MDD^1^					
	*n*	%	*M*	*SD*	*n*	%	*M*	*SD*
Biological Sex								
Female	49	64.4			30	48.4		
Male	27	35.6			32	51.6		
Age	76		66.36	7.07	62		64.89	5.73
Education	76		16.99	2.39	61		16.44	2.38
MADRS^2^	74		1.88***	2.01	62		15.11	11.68

^1.^Major Depressive Disorder; ^2.^Montgomery Asperg Depression Rating Scale.

***p <.001.

### Procedures

All study procedures were approved by the Institutional Review Board of each respective study site. Participants were initially screened over the phone with a comprehensive set of questions that assessed whether they met basic inclusion/exclusion criteria for the study. Phone screen questions appraised their ability to engage in neuropsychological testing (i.e., having sufficient visual and hearing capacity to follow instructions, fluency in the English language, absence of color blindness) and an evaluation of their overall health. Eligible participants had no history of serious medical conditions (i.e., metastatic cancer, brain tumors, unstable cardiac, hepatic, or renal disease, myocardial infarction or stroke) or untreated chronic medical illnesses (i.e., diabetes, hypertension) within the three months preceding the study, any serious neurological disorders (i.e. Parkinson’s Disease, MS, epilepsy), major physiological disturbance or major chronobiological disruption or phase shift during the preceding month, autoimmune disease, serious head trauma, nutritional problems, surgery, trauma, developmental disabilities, sleep apnea, active abuse of substances in the 6 months preceding the study, or an organic brain syndrome such as depression due to closed head injury, as well as any conditions often associated with depression. Participants with an active suicide plan, or those who were taking any drugs causing depression (i.e., steroids, αmethyl-dopa, clonidine, reserpine, tamoxifen, or cimetidine), or who were unable to abstain from taking benzodiazepine, opioid or psychostimulant medications were also excluded. Because the study involved participants undergoing an MRI scan, those with contraindications for MRI (i.e., having metallic implants that are not MRI safe, claustrophobia, or pacemakers) were not included. Participants with depression, in addition to the overall inclusion criteria, had to have their first onset of depression prior to the age of 35. Healthy comparisons were required to have no history of psychiatric illness in themselves or a first-degree relative. Participants were also excluded from this study if they met Jak & Bondi psychometric criteria for amnestic Mild Cognitive Impairment (28).

Eligible participants underwent standard IRB-approved informed consent procedures and were then invited to undergo diagnostic screening, including a structured clinical interview using the Structured Clinical Interview for DSM-5-Research Version (29) to assess group membership (depression or control) and rule out bipolar disorder, psychosis, and other Axis I psychiatric diagnoses (excluding anxiety disorders). Participants also completed the Montgomery-Asberg Depression Rating Scale (MADRS), a semi-structured interview to assess depression symptom severity (30). Those with estimated premorbid cognitive functioning of less than 80 (as assessed by the Wide Range Achievement Test-4) were excluded. Following diagnostic screening, participants underwent a comprehensive neuropsychological assessment and completed self-report questionnaires.

### Measures

Personality was assessed using the NEO Personality Inventory-Revised (NEO-PI-R), a standard measure of the Five Factor Model of personality (2). Respondents were asked to rate the extent to which they agree with 240 statements using a 5-point scale (strongly disagree–strongly agree). All participants completed a neuropsychological battery consisting of measures described in **Table 2**. Participants completed the measures on paper or electronically. Outcome variables included verbal learning and memory, visual learning and memory, fine motor dexterity, and multiple aspects of executive functioning (i.e., set shifting, cognitive flexibility, problem solving, phonemic verbal fluency, and semantic verbal fluency). Study materials, such as the protocol and analytic methods, are available upon request.

**Table 2:** Neuropsychological Measures.

Measure	Description	Cognitive Domain Measured
**Hopkins Verbal Learning Test-Revised (HVLT)**	The HVLT is a widely used auditory measure of word list learning and memory that consists of three learning trials of a 12-item list, followed by 20- to 25-minute delayed recall and recognition tests (31).	Auditory Memory
**Weschler Memory Scale-IV (WMS-IV) Logical Memory Test**	The Logical Memory Test is a standardized measure of verbal memory where participants are read two stories and are then asked to immediately recall everything they can from the stories, using as many details as possible in their response. After a 20-30 minute delay, they are again asked to recount as much information from the stories as they can remember. Following the delay trial, the subject is asked specific ‘yes’ or ‘no’ questions about the stories, testing whether they are able to recognize aspects of the stories (32).	Auditory Memory
**Brief Visuospatial Memory Test-R (BVMT-R)**	The BVMT-R is a widely used and accepted measure that consists of three learning trials during which the subject is presented with a display containing six figures to study for ten seconds and is then asked to recreate the display from their memory. The three trials are followed by a 20–25-minute delay period, after which the participant is asked to again draw the display from their memory, and is then administered a recognition test (33).	Visual memory
**Purdue Pegboard Test**	This task requires the examinee to place as many pins as they can into holes on a pegboard in 30 seconds, first using their right hand, then their left, and then both hands simultaneously (34). This task has been used extensively to measure fine motor skills and gross movements.	Fine Motor Dexterity
**Trail Making Tests A & B**	Trail Making Test A is a timed task that requires participants to draw a line connecting numbers in increasing order as quickly as they can without making errors or lifting the pen from the paper. In Trail Making Test B, the participant must alternate between connecting numbers and letters in increasing order, again working as fast as they can (35).	Attentional Shifting
**Wisconsin Card Sorting Test (WCST)**	The WCST is one of the most widely used and accepted measure of executive function. During this task, participants are asked to match 128 cards to one of four key cards, based on a rule that is unknown to the examinee, but that can be surmised based upon examiner feedback. They are told each time whether they are right or wrong, and to leave the card where placed and try to get the next response next (36).	Cognitive Flexibility
**Delis Kaplan Executive Function System (D-KEFS) Sorting Test**	The DKEFS Sorting Test asks subjects to sort a set of cards into groups by categories that they must generate using properties of the cards. The objective of this task is for the participant to come up with as many distinct categories as possible, and to accurately explain how they classified the cards in that particular manner (37)	Reasoning/Problem Solving
**Controlled Oral Word Association Task – F-A-S**	The COWAT FAS requires examinees to name as many words as they can that begin with a certain letter (“F”, “A”, and “S”) in one minute (38).	Phonemic Fluency
**Animal Naming Task**	During the Animal Naming task, participants are asked to name as many animals as they can think of in one minute (39).	Semantic Fluency

### Data analysis

SPSS 28.0.1.1 (14) was used for all analyses. To measure auditory memory, visual memory, and fine motor dexterity, we created summary scores of measures reflecting performance in each domain. Cognitive outcome measures and internal consistency values for each of these domains are included in **Supplementary Table 1**. Summary scores for each cognitive domain were calculated by first, converting raw scores for each test within a domain to a whole sample-based z-score, then averaging the resulting z-scores within each domain. As executive functioning is a complex domain that sometimes measures disparate cognitive skills, measures reflecting executive functioning were considered separately [i.e., Delis-Kaplan Executive Function System (D-KEFS) Confirmed Correct Sorts, Wisconsin Card Sorting Test (WCST) Perseverative Responses, Trail Making Test Part A minus Part B, Animal Naming, FAS Phonemic Fluency). All raw scores were converted to sample z-scores for ease of interpretation. NEO-PI T scores (using manualized normative data) were used in all analyses.

Bivariate Pearson’s correlations were conducted to assess relationships between each cognitive factor score and the five personality factor scores. For significant correlations, a series of linear regression models were performed. Age, education and biological sex were entered into Block 1, depression status (never vs. ever) was entered in Block 2, and the personality factor score was entered in Block 3. When full models and the factor score were significant, another series of hierarchical regression models with the sub-facet scores entered in step 3 were performed. Significance was set at α = .05 for all analyses.

## Results

Pearson correlation analyses revealed significant relationships between Openness and the Brief Visuospatial Memory Test- Revised (*r* = .18, *p* <.05), WCST Perseverative Responses (*r* = -.29, *p* <.01), Animal Naming (*r* = .23, *p* <.01), and FAS Phonemic Fluency (*r* =.21, *p* <.05). No other significant relationships between cognitive performance and personality factors were found (see **Supplementary Table 2**).

When demographic variables and depression status were included in hierarchical regression analyses, relationships between these cognitive variables and Openness remained significant (see **Supplementary Tables 3–6**).

In the final set of analyses, we sought to further consider the sub-facets of openness that are related to executive functioning. After accounting for demographic variables and depression status in Blocks 1 and 2, respectively, all sub-facet scores were entered in the final model. We found that *Openness to Values* positively predicted cognitive flexibility (*B* = - 0.31, *p* <.01; **Table 3a**) and semantic fluency (*B* = 0.21, *p* <.05; **Table 3b**). *Openness to Feelings* and *Openness to Values* were positively related to phonemic fluency (*B* = 0.30, *p* <.01; *B* = 0.27, *p* <.01) (**Table 3c**). None of the sub-facet scores were significant in the final model for visual memory. Other openness sub-facets, including *Openness to Fantasy, Aesthetics, Actions, and Ideas*, were not significant. Importantly, when depression symptom severity (i.e., MADRS score) was added to Step 2 in all models, results did not change. Thus, results presented consider only current or past history of depression.

**Table 3A:** Full Regression Model Predicting Cognitive Flexibility^1^.

Predictors	*B^2^*	*SE*	*t*	*F*	*R* ^2^
*Block 1*				2.60**	.19
Age	-.15	.01	1.65		
Sex	.12	.20	1.19		
Education	-.05	.04	-.61		
*Block 2*					
Depression Status	-.00	.20	-.02		
*Block 3*					
*(Openness to Experience Subfacets)*					
Fantasy	-.50	.03	-1.47		
Aesthetics	.32	.04	.97		
Feelings	.07	.01	.66		
Actions	.03	.01	.28		
Ideas	-.02	.01	-.15		
Values	-.31**	.01	-3.06		

** p< .01.

**Table 3B:** Full Regression Model Predicting Semantic Fluency.

Predictors	*B^2^*	*SE*	*t*	*F*	*R* ^2^
*Block 1*				3.95***	.24
Age	-.28***	.01	-3.42		
Sex	-.10	.19	-1.11		
Education	.22**	.04	2.68		
*Block 2*					
Depression Status	-.05	.18	-.59		
*Block 3*					
*(Openness to Experience Subfacets)*					
Fantasy	.12	.03	.41		
Aesthetics	.04	.03	.12		
Feelings	.10	.01	.99		
Actions	-.10	.01	-1.10		
Ideas	-.02	.01	-.24		
Values	.21*	.01	2.24		

*p < .05, **p< .01, ***p ≤ .001.

**Table 3C:** Full Regression Model Predicting Phonemic Fluency.

Predictors	*B^2^*	*SE*	*t*	*F*	*R* ^2^
*Block 1*				4.78***	.28
Age	-.21*	.01	-2.61		
Sex	.15	.18	1.64		
Education	.23**	.03	2.84		
*Block 2*					
Depression Status	-.12	.17	-1.39		
*Block 3*					
*(Openness to Experience Subfacets)*					
Fantasy	-.46	.03	-1.59		
Aesthetics	.35	.03	1.22		
Feelings	.30**	.01	3.13		
Actions	-.05	.01	-.58		
Ideas	.02	.01	.22		
Values	.27**	.01	3.07		

^*1.^Higher scores indicate poorer performance. ^2.^Standardized coefficient.

*p < .05, **p< .01, ***p ≤ .001.

## Discussion

This study uniquely measured the relationship between cognition and personality in older individuals with onset of depression prior to middle age. Results indicated that Openness to Experience is positively associated with aspects of executive functioning (i.e., verbal fluency and cognitive flexibility) and to a smaller extent, visual learning and memory. Relationships between executive functioning measures were driven by a significant association between two facets of openness: *Openness to Values* and *Openness to Feelings*. These results supported our hypothesis that openness to experience would positively predict cognitive performance. In contrast, our results did not support a relationship between neuroticism and cognitive functioning.

*Openness to Values* is defined as the willingness to reassess social, political, and religious values. Individuals scoring high on openness to values may be more flexible and have a less rigid mindset, which may be advantageous on cognitive flexibility tasks such as the WCST. People who score higher on the *Openness to Feelings* facet tend to consider emotions a valuable part of the human experience and are more attuned to their feelings. They often experience emotions more deeply than those scoring lower. Emotions and executive functioning have been found to be highly related, with intact executive functioning an essential component to the deployment of successful emotion regulation strategies (40). Effective deployment of emotion regulation strategies is dependent on intact executive functioning skills, including cognitive control and problem solving, and involves the efficacious engagement of the dorsolateral prefrontal cortex and ventral frontal cortex (41). The significant correlation between *Openness to Feelings* and performance on a phonemic fluency task—an aspect of executive functioning—may be a manifestation of the interrelatedness of emotion regulation and executive function.

Our results are consistent with multiple studies that found a positive correlation between *Openness to Experience* and performance on tasks of verbal fluency (9, 15, 17, 18, 42) and executive functioning (24, 42). Ayotte and colleagues (20) studied older adults with depression and did not find a significant association between Openness and phonemic fluency performance. They did, however, observe a positive association between Openness and other aspects of executive functioning in participants with a history of depression relative to non-depressed participants. Given the lack of literature on personality and cognition in older adults with a history of depression, we do not have an empirical explanation for the disparity between our findings and Ayotte and colleagues. Our results also diverge from Booth and colleagues (14) who found a positive association between Openness and verbal memory but failed to detect a significant correlation between Openness and tasks of executive function. Notably, this study examined the effects of personality and state depression and anxiety on cognition, though participants were not screened for psychiatric disorders, rendering depression diagnoses unknown. As their sample was older and were subjected to less stringent exclusion criteria, this may explain the differences. Our observation that openness was positively associated with visual memory adds a novel finding to the literature, which warrants further study.

Our expectation that neuroticism would be negatively associated with performance on tasks of episodic memory and executive functioning was unsupported, contributing to an already equivocal literature. Previous evidence parallels this negative correlation in a similar demographic (16). Yet, in a slightly older sample, Sutin et al. (13) found an inverse relationship between neuroticism scores and cognitive tasks across several domains. Similarly, Boyle et al. (3) found a significant negative relationship between neuroticism and global cognitive function (measured by MMSE). When looking at executive functioning specifically, their results did not hold. The Da Silva et al. study (4) provides evidence for neuroticism as a negative corollary with executive functioning in an older group, though they identified perceived stress as an explanatory mediator. Mirroring these results, Chapman and colleagues (8) found higher neuroticism associated with poorer cognitive functioning. Taken together, the age in these samples averaged a decade older. Although Soubelet and Salthouse (17) found that cognition–personality associations did not differ significantly among different age groups in adulthood, a more recent study supports that facets of personality are associated with cognition differentially by age (9). Further study on the personality–cognition relationship, focusing on the facet-level interactions, is needed to elucidate whether differences in age between samples may explain the disparity between our findings and those of other studies. Importantly, our sample comprised both healthy controls and individuals with a lifetime history of depression. Given that depression is strongly associated with neuroticism (3), it is possible that any association between cognition and neuroticism is better explained by the presence of trait depression in nearly half the sample.

Finally, our findings that the other three factors of personality (conscientiousness, agreeableness, and extraversion) were not significantly associated with any of the cognitive domains or measures is unsurprising given the mixed literature. Still, further research exploring these relationships in older adults with a history of depression would be beneficial, as research on this topic is scarce.

Findings should be interpreted in the context of study limitations. The sample was predominantly White with high levels of educational achievement (and presumably cognitive reserve), which limits generalizability. We also only explored the facets of openness and their relation to cognition, though cognition can be related to certain facets of a trait without having a significant association with the overall trait (9). Furthermore, the cross-sectional design of this investigation limits our ability to draw conclusions about trends in the personality–cognition relationship over time.

Our study corroborates prior studies that found positive relationships between Openness to Experience and multiple domains of cognition in older adults. Future research should explore interventions that can facilitate openness and test their efficacy in promoting or preserving cognition. Further study is needed to better understand the relationship between personality, cognition, and depression in older adults, and especially to elucidate the bi-directional nature of these relationships. Longitudinal studies investigating this relationship over time may lend important insight into whether certain personality traits may be a risk factor for cognitive decline or contribute to cognitive reserve in aging adults with major depressive disorder. In an applied setting, implementing personality assessments during initial sessions with older adults with depression may help to identify persons that are at risk for cognitive decline and would benefit from early intervention. Using personality assessment and developing patient-specific interventions based on their personality profile may also improve older patients’ treatment adherence and outcomes (1). In a broader social context, it would be interesting to explore whether socially-oriented interventions that provide education around a range of perspectives and exposure to new experiences could function to bolster executive functioning in older adults.

This study analyzed the specific sub-facets underlying the significant relationship between Openness to Experience and executive functioning, whereas most studies focused on the associations at the factor level. A strength of our study is our use of stringent exclusionary criteria and that all participants underwent structured psychodiagnostics interviewing to determine depression diagnosis rather than using a self-report scale. We also used a comprehensive neuropsychological test battery to evaluate cognition to ensure sampling of each of the major cognitive domains.

Understanding the relationship between personality and cognition can help identify older adults who may be at greater risk for cognitive decline and inform intervention approaches. Our results suggest that older people who are more capable of experiencing, identifying, and understanding feelings within themselves and others have stronger executive functioning. This finding also emerged in those who are more willing to re-examine social, political, and religious values. Psychotherapies and other behavioral interventions that facilitate open-mindedness in older adults may have a parallel impact on improving executive functioning, though this would need to be examined prospectively.

## Data availability statement

The original contributions presented in the study are included in the article/**Supplementary Material** Further inquiries can be directed to the corresponding author/s.

## Ethics statement

The studies involving humans were approved by Stony Brook University IRB Human Research Protection Program. The studies were conducted in accordance with the local legislation and institutional requirements. The participants provided their written informed consent to participate in this study.

## Author contributions

MA: Writing – original draft, Project administration, Conceptualization. JKa: Writing – review & editing, Writing – original draft, Project administration. JM: Writing – review & editing, Writing – original draft. DS: Writing – review & editing, Formal analysis. JKi: Writing – review & editing, Project administration. VK: Writing – review & editing. SL: Writing – review & editing. SW: Writing – review & editing, Writing – original draft, Supervision, Methodology, Investigation, Funding acquisition, Formal analysis, Data curation, Conceptualization.
