# Supplementary material for: The relationship between personality and cognition in older adults with and without early-onset depression
Source: Front Psychiatry. 2024 Jul 10;15:1337320. doi: 10.3389/fpsyt.2024.1337320 (PMC11266124; doi:10.3389/fpsyt.2024.1337320)
Supplement: Supplementary file 1 [file Table_1.docx]

| Cognitive Domain | Outcome Measures | Cronbach’s Alpha |
| --- | --- | --- |
| Auditory Memory | HVLT-R^1^ Recall 1-3  HVLT-R^1^ Delayed Recall  Logical Memory I  Logical Memory II | .85 |
| Visual Memory | BVMT-R^2^ Recall 1-3  BVMT-R^2^ Delayed Recall | .89 |
| Fine Motor Dexterity | Purdue Pegboard Dominant  Purdue Pegboard Non-Dominant  Purdue Pegboard Bimanual | .81 |
| Attentional Shifting | Trails B-A | NA |
| Cognitive Flexibility | WCST^3^ Perseverative Responses | NA |
| Reasoning/Problem Solving | D-KEFS^4^ Confirmed Correct Sorts | NA |
| Phonemic Fluency | COWAT^5^ (FAS) | NA |
| Semantic Fluency | Animal Naming Total Correct | NA |
| *Note.* 1. Hopkins Verbal Learning Test-Revised (34); 2. Brief Visuospatial Memory Test-Revised (36); 3. Wisconsin Card Sorting Test (39); 4. Delis Kaplan Executive Function System (40); 5. Controlled Oral Word Association Test (41) | | |

Supplemental Table 1.

*Internal Consistency of Measured Cognitive Domains*
