# Supplementary material for: The relationship between personality and cognition in older adults with and without early-onset depression
Source: Front Psychiatry. 2024 Jul 10;15:1337320. doi: 10.3389/fpsyt.2024.1337320 (PMC11266124; doi:10.3389/fpsyt.2024.1337320)
Supplement: Supplementary file 2 [file Table_2.docx]

| Supplemental Table 2  *Pearson Correlations Between Cognitive Domains and NEO-PI Five Traits* | | | | | | | | | | | | | |
| --- | --- | --- | --- | --- | --- | --- | --- | --- | --- | --- | --- | --- | --- |
|  | Aud Mem | Vis Mem | Fine Mot | Trails B-A | WCST Pers  Resp | D-KEFS Sort | Animal Naming | COWAT | Neuroticism | Extroversion | Openness | Agreeableness | Conscientiousness |
| Aud Mem  Vis Mem  Fine Mot  Trails B-A  WCST Pers Resp^1^  D-KEFS Sorting  Animal Naming  COWAT  Neuroticism  Extroversion  Openness  Agreeableness  Conscientiousness |  |  |  |  |  |  |  |  |  |  |  |  |  |
|  | .40^**^ |  |  |  |  |  |  |  |  |  |  |  |  |
|  | .23^*^ | .39^**^ |  |  |  |  |  |  |  |  |  |  |  |
|  | -.31^**^ | -.26^**^ | -.32^**^ |  |  |  |  |  |  |  |  |  |  |
|  | -.27^**^ | -.20^*^ | -.17 | .28^**^ |  |  |  |  |  |  |  |  |  |
|  | .23^*^ | .21^*^ | .30^**^ | -.18^*^ | -.19^*^ |  |  |  |  |  |  |  |  |
|  | .37^**^ | .29^**^ | .19^*^ | -.26^**^ | -.22^*^ | .29^**^ |  |  |  |  |  |  |  |
|  | .25^**^ | .19^*^ | .02 | -.25^**^ | -.15 | .31^**^ | .46^**^ |  |  |  |  |  |  |
|  | -.02 | .04 | -.08 | .11 | .10 | .11 | .09 | .03 |  |  |  |  |  |
|  | .00 | -.15 | -.00 | -.14 | .02 | -.11 | -.04 | .09 | -.33^**^ |  |  |  |  |
|  | .10 | **.18^*^** | .03 | -.07 | **-.29^**^** | .18 | **.23^**^** | **.21^*^** | .06 | -.11 |  |  |  |
|  | .06 | .06 | .06 | .09 | .03 | -.03 | -.13 | -.15 | .20^*^ | -.00 | -.14 |  |  |
|  | -.08 | -.13 | .00 | -.08 | .06 | -.06 | -.01 | .03 | -.31^**^ | .12 | .02 | -.36^**^ |  |
| *Note.* 1. Higher scores are indicative of poorer performance. | | | | | | | | | | | | | |
| **. Correlation is significant at the 0.01 level (2-tailed). | | | | | | | | | | | | | |
| *. Correlation is significant at the 0.05 level (2-tailed). | | | | | | | | | | | | | |
