# Supplementary material for: The relationship between personality and cognition in older adults with and without early-onset depression
Source: Front Psychiatry. 2024 Jul 10;15:1337320. doi: 10.3389/fpsyt.2024.1337320 (PMC11266124; doi:10.3389/fpsyt.2024.1337320)
Supplement: Supplementary file 4 [file Table_4.docx]

| Supplemental Table 3b  *Full Regression Model Predicting Cognitive Flexibility*^1^ | | | | | |
| --- | --- | --- | --- | --- | --- |
| **Predictors** | ***B*^2^** | ***SE*** | ***t*** | ***F*** | ***R*^2^** |
| *Block 1* |  |  |  | 3.30** | .12 |
| Age | .18* | .01 | 2.03 |  |  |
| Sex | .08 | .18 | .95 |  |  |
| Education | -.09 | .04 | -1.07 |  |  |
| *Block 2* |  |  |  |  |  |
| Depression Status | -.01 | .18 | -.11 |  |  |
|  |  |  |  |  |  |
| *Block 3* |  |  |  |  |  |
| NEO-PI Openness | -.28** | .01 | -3.20 |  |  |

*Note.* 1. Higher scores are indicative of poorer performance; 2. standardized coefficient.

^*^ *p* < .05, ^**^ *p*< .01, ^***^ *p* ≤ .001.
