# Supplementary material for: The relationship between personality and cognition in older adults with and without early-onset depression
Source: Front Psychiatry. 2024 Jul 10;15:1337320. doi: 10.3389/fpsyt.2024.1337320 (PMC11266124; doi:10.3389/fpsyt.2024.1337320)
Supplement: Supplementary file 5 [file Table_5.docx]

| Supplemental Table 3c  *Full Regression Model Predicting Semantic Fluency* | | | | | |
| --- | --- | --- | --- | --- | --- |
| **Predictors** | ***B*^1^** | ***SE*** | ***t*** | ***F*** | ***R*^2^** |
| *Block 1* |  |  |  | 6.52*** | .20 |
| Age | -.31*** | .01 | -3.90 |  |  |
| Sex | -.10 | .16 | -1.18 |  |  |
| Education | .22** | .03 | 2.71 |  |  |
| *Block 2* |  |  |  |  |  |
| Depression Status | -.01 | .16 | -.12 |  |  |
|  |  |  |  |  |  |
| *Block 3* |  |  |  |  |  |
| NEO-PI Openness | .23** | .01 | 2.85 |  |  |

*Note.* 1. standardized coefficient.

^*^ *p* < .05, ^**^ *p*< .01, ^***^ *p* ≤ .001.
