# Supplementary material for: The relationship between personality and cognition in older adults with and without early-onset depression
Source: Front Psychiatry. 2024 Jul 10;15:1337320. doi: 10.3389/fpsyt.2024.1337320 (PMC11266124; doi:10.3389/fpsyt.2024.1337320)
Supplement: Supplementary file 6 [file Table_6.docx]

| Supplemental Table 3d  *Full Regression Model Predicting Phonemic Fluency* | | | | | |
| --- | --- | --- | --- | --- | --- |
| **Predictors** | ***B*^1^** | ***SE*** | ***t*** | ***F*** | ***R*^2^** |
| *Block 1* |  |  |  | 4.78*** | .16 |
| Age | -.24** | .01 | -2.89 |  |  |
| Sex | .04 | .17 | .51 |  |  |
| Education | .23** | .04 | 2.79 |  |  |
| *Block 2* |  |  |  |  |  |
| Depression Status | -.05 | .17 | -.58 |  |  |
|  |  |  |  |  |  |
| *Block 3* |  |  |  |  |  |
| NEO-PI Openness | .18* | .01 | 2.18 |  |  |

*Note.* 1. standardized coefficient.

^*^ *p* < .05, ^**^ *p*< .01, ^***^ *p* ≤ .001.
